# Clinical value of dark-blood late gadolinium enhancement cardiovascular magnetic resonance without additional magnetization preparation

**DOI:** 10.1186/s12968-019-0556-1

**Published:** 2019-07-29

**Authors:** Robert J. Holtackers, Caroline M. Van De Heyning, Muhummad Sohaib Nazir, Imran Rashid, Ioannis Ntalas, Haseeb Rahman, René M. Botnar, Amedeo Chiribiri

**Affiliations:** 1 Department of Radiology, Maastricht University Medical Centre, Maastricht, the Netherlands; 2 Department of Cardiovascular Imaging, School of Biomedical Engineering and Imaging Sciences, King’s College London, 4th Floor, Lambeth Wing, St Thomas’ Hospital, London, SE1 7EH UK; 3 Department of Cardiology, St Thomas’ Hospital, London, UK; 4 Department of Cardiology, Antwerp University Hospital, Edegem, Belgium; 5 Cardiovascular Diseases, University of Antwerp, Antwerp, Belgium; 6 Escuela de Ingeniería, Pontificia Universidad Católica de Chile, Santiago, Chile; 7 CARIM School for Cardiovascular Diseases, Maastricht University, Maastricht, the Netherlands

**Keywords:** Late enhancement, Late gadolinium enhancement, LGE, Dark-blood, Myocardial scar, Subendocardial scar

## Abstract

**Background:**

For two decades, bright-blood late gadolinium enhancement (LGE) cardiovascular magnetic resonance (CMR) has been considered the reference standard for the non-invasive assessment of myocardial viability. While bright-blood LGE can clearly distinguish areas of myocardial infarction from viable myocardium, it often suffers from poor scar-to-blood contrast, making subendocardial scar difficult to detect. Recently, we proposed a novel dark-blood LGE approach that increases scar-to-blood contrast and thereby improves subendocardial scar conspicuity. In the present study we sought to assess the clinical value of this novel approach in a large patient cohort with various non-congenital ischemic and non-ischemic cardiomyopathies on both 1.5 T and 3 T CMR scanners of different vendors.

**Methods:**

Three hundred consecutive patients referred for clinical CMR were randomly assigned to a 1.5 T or 3 T scanner. An entire short-axis stack and multiple long-axis views were acquired using conventional phase sensitive inversion recovery (PSIR) LGE with TI set to null myocardium (bright-blood) and proposed PSIR LGE with TI set to null blood (dark-blood), in a randomized order. The bright-blood LGE and dark-blood LGE images were separated, anonymized, and interpreted in a random order at different time points by one of five independent observers. Each case was analyzed for the type of scar, per-segment transmurality, papillary muscle enhancement, overall image quality, observer confidence, and presence of right ventricular scar and intraventricular thrombus.

**Results:**

Dark-blood LGE detected significantly more cases with ischemic scar compared to conventional bright-blood LGE (97 vs 89, *p* = 0.008), on both 1.5 T and 3 T, and led to a significantly increased total scar burden (3.3 ± 2.4 vs 3.0 ± 2.3 standard AHA segments, *p* = 0.015). Overall image quality significantly improved using dark-blood LGE compared to bright-blood LGE (81.3% vs 74.0% of all segments were of highest diagnostic quality, *p* = 0.006). Furthermore, dark-blood LGE led to significantly higher observer confidence (confident in 84.2% vs 78.4%, *p* = 0.033).

**Conclusions:**

The improved detection of ischemic scar makes the proposed dark-blood LGE method a valuable diagnostic tool in the non-invasive assessment of myocardial scar. The applicability in routine clinical practice is further strengthened, as the present approach, in contrast to other recently proposed dark- and black-blood LGE techniques, is readily available without the need for scanner adjustments, extensive optimizations, or additional training.

## Background

Although coronary artery disease related deaths have declined over the past 10 years, it remains the leading cause of death worldwide for both men and women [1]. Since survivors of previous myocardial infarction (MI) face an increased risk of new cardiovascular events, there is great focus on detecting and accurately assessing the infarcted region [2]. Important aspects of this assessment include determining the infarct’s location, size, and transmural extent. Over a decade ago it was first demonstrated that even small regions of MI were associated with large increases in major adverse cardiac events [3, 4], while regional functional recovery following revascularization was highly dependent on the transmural extent of the infarct [5–8]. It is clear that this information is an essential requisite for patient management decisions and selection of the optimal therapeutic approach.

Bright-blood late gadolinium enhancement (LGE) cardiovascular magnetic resonance (CMR) has been considered the reference standard in the non-invasive assessment of myocardial viability for almost two decades now. Its ability to clearly depict areas of myocardial infarction from viable myocardium is well established, making LGE a widely accepted component of standard clinical CMR protocols. By nulling the magnetization level of viable myocardium, its dark appearance can easily be distinguished from the bright appearance of scar tissue. However, as the adjacent LV blood pool can have similar T1 values at 10 min post-injection with almost equally bright signal, the border between scar and blood can be difficult to delineate. In particular patients with thin subendocardial scarring patterns in this border zone are susceptible to this limitation, where the apparent volume of scar tissue can be significantly reduced, or even completely obscured. Furthermore, blood pool signal can mimic scar tissue and lead to false positive observations. This makes subendocardial scar patterns difficult to detect and clearly delineate using conventional bright-blood LGE. Allowing additional time after contrast administration partly resolves this challenge, as due to contrast washout over time the scar and blood T1 values will start to diverge, thereby increasing scar-to-blood contrast. However, this solution is unfavourable in daily clinical practice, where already long CMR examinations and high clinical demand result in significant time-pressures.

Since the initial validation against histology in 1999 [9], the clinical utility of LGE has broadened and several variations in the technique’s application have been introduced. These advances include new reconstruction techniques [10–14], free-breathing sequences using motion correction algorithms [15–18], and techniques to improve image contrast, in particular the poor scar-to-blood contrast [18–26]. These techniques improve scar-to-blood contrast by using additional magnetization preparation before or after the 180° inversion pulse. Multiple approaches have been proposed, including T_2_ preparation [18–23], magnetization transfer [23, 24], T_1_ rho using spin locking [24], and multiple subsequent inversion pulses [25, 26], each creating a different type of contrast and improving subendocardial scar conspicuity. However, the use of additional magnetization preparation mechanisms often requires adjustments to the scanner software, extensive optimizations for new sequence parameters, additional training for radiographers, and most importantly, are not readily available in routine clinical practice. Furthermore, specific absorption rate levels are increased due to the additional radiofrequency pulses.

Recently, we proposed a novel dark-blood LGE approach that significantly increases scar-to-blood contrast without using any additional magnetization preparation mechanism [27]. In a small pilot study performed on patients with previous MI, nulling the LV blood pool instead of the myocardium (by choosing a shorter TI), in combination with phase-sensitive inversion-recovery (PSIR), has shown to provide clear benefits for the identification of subendocardial scar. These experiments demonstrated that this novel dark-blood LGE technique has a significantly higher scar-to-blood contrast-to-noise ratio (CNR) than conventional bright-blood LGE, while maintaining the scar-to-myocardium CNR. In the present work we sought to assess the clinical value of this novel dark-blood approach for the detection of ischemic injury in a large cohort of patients on both 1.5 and 3 T, in comparison with reference standard bright-blood LGE imaging. As no additional magnetization preparation mechanisms are required, the novel approach is readily available in routine clinical setting without the need for scanner software adjustments, extensive parameter optimizations, and additional training.

## Methods

### Study population

A total of 300 consecutive patients referred for clinical CMR including LGE between March and September 2017 were included. Patients were randomly allocated to either a 1.5 T or 3 T clinical CMR scanner of different vendors: 1.5 T Ingenia (Philips Healthcare, Best, the Netherlands) [*n* = 100], 1.5 T Aera (Siemens Healthineers, Erlangen, Germany) [n = 100], and 3 T Achieva TX (Philips Healthcare) [n = 100]. The study was approved by the local ethics committee (15/NS/0030) and was conducted according to the Declaration of Helsinki. Written informed consent was obtained from all patients for inclusion in the study and for additional CMR imaging during their clinical CMR exam.

### LGE imaging

**Fig. 1:**
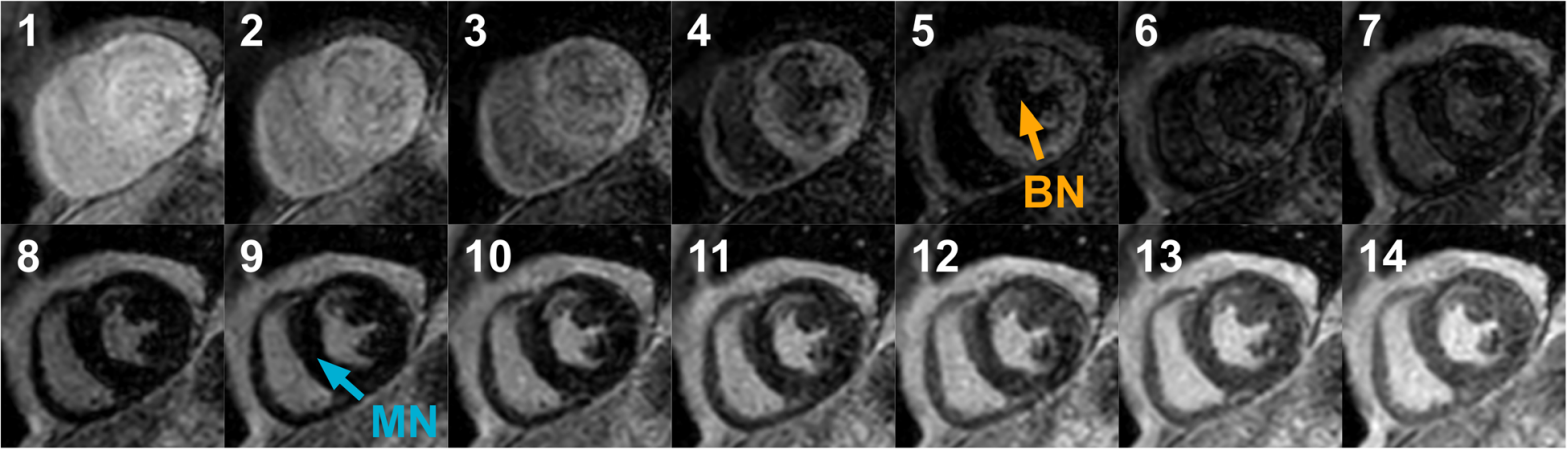
A series of short-axis images with varying inversion times (TI) (Look-Locker scan) in a subject with myocardial scar, acquired at ten minutes post-injection. The blood pool, viable myocardium, and myocardial scar each reach their own nulling point at different TIs. Note that for conventional bright-blood late gadolinium enhancement (LGE) the TI is set to null viable myocardium (blue arrow in frame 9, TI = 248 ms), while for dark-blood LGE the TI is set to null the blood pool (orange arrow in frame 5, TI = 150 ms)

A routine clinical CMR protocol was used to obtain a cine stack of the short-axis view covering the entire left ventricle (LV) and right ventricle (RV) (10–15 slices), followed by cine images of the two-, three-, and four-chamber view. After an intravenous injection of 0.2 mmol/kg gadobutrol (Gadovist, Bayer, Berlin, Germany), both conventional bright-blood LGE and proposed dark-blood LGE images were acquired in identical cardiac projections (full short-axis stack covering the entire LV and RV + three long-axis views). A 1:1 randomisation scheme was used to decide the order of acquisition of the LGE images. In 150 consecutive subjects (50 for each CMR scanner), conventional bright-blood LGE was performed first (starting at 10 min post-injection), followed by dark-blood LGE (starting at 20 min post-injection). In another 150 consecutive subjects (50 for each CMR scanner), the scans were acquired in reversed order: dark-blood LGE first (10 min post-injection), followed by conventional bright-blood LGE (20 min post-injection). The mechanism for the dark-blood LGE acquisition technique without using additional magnetization preparation has recently been described in detail [27]. Both methods used a PSIR acquisition and reconstruction, preceded by a Look-Locker sequence to determine the TI. For conventional bright-blood LGE, the TI was set to null viable LV myocardium, while for dark-blood LGE the TI was set to null the LV blood pool (Fig. 1), leading to a dark-gray appearance of the blood in the PSIR image. Specific sequence details for all three CMR scanners reflect the local clinical protocol and can be found in Table 1. Note that only the TI was adjusted in the local clinical protocols to acquire dark-blood LGE images. All images were acquired in the mid-diastolic resting period using 10–15 s breath-hold scans, depending on the subject’s heart rate. The given contrast dose reflects local protocol and current international guidelines [28].

**Table 1:** Phase-sensitive inversion-recovery LGE pulse sequence parameters

	Philips Ingenia	Siemens Aera	Philips Achieva TX
	*(n = 100)*	*(n = 100)*	*(n = 100)*
*Field strength (T)*	1.5	1.5	3.0
*Readout type*	T_1_-TFE	TrueFISP	T_1_-TFE
*Echo time (TE, ms)*	3.0	1.2	2.0
*Repetition time (TR, ms)*	6.2	2.9	3.6
*Flip angle (°)*	25	45	25
*Slice thickness (mm)*	10	8	8
*Acquired resolution (mm* ^*2*^ *)*	1.60 × 1.90	1.41 × 1.89	1.61 × 1.61

### Image analysis

The acquired image data of all subjects was randomly assigned for review to one of five independent level III trained observers blinded to subject characteristics. For each subject, the conventional bright-blood and dark-blood PSIR LGE images were separated, anonymized, and analyzed in random order at different time points. Thereby, the bright-blood and dark-blood LGE images of the same subject were never presented in sequence to the reader. The images were shown and interpreted according to the criteria recommended by the Society for Cardiovascular Magnetic Resonance (SCMR) [29]. All images were analysed using OsiriX v9.0 64-bit (Pixmeo, Geneva, Switzerland).

Overall image quality was rated on a four-point Likert scale for each method: 0) non-diagnostic, 1) low [≥ two segments non-diagnostic], 2) medium [one segment non-diagnostic], or 3) high [all segments correctly identified]). In case the image quality of either of the two methods was scored as 0 (non-diagnostic), the subject was excluded from further analysis. Subjects were classified with each LGE method as having: 0) no scar, 1) ischemic scar, 2) non-ischemic scar, or 3) both ischemic and non-ischemic LV scar. In case the classifications, as assigned by each method, did not match, consensus was provided by a level III observer with > 10 years of experience in CMR. In case any ischemic scar was observed, the entire short-axis stack was analyzed for maximum scar transmurality on a per-segment basis using the American Heart Association (AHA) model (17 segments). The transmural extent in each segment was evaluated as a percentage of the local total wall thickness using a five-point Likert scale: 0) no scar, 1) 1–25%, 2) 26–50%, 3) 51–75%, or 4) 76–100%. Total scar burden was calculated as the sum of all segments multiplied by their corresponding maximum transmurality percentage (maximum scar burden = 17). Observer confidence in scar detection and analysis was rated using a binary scale: 0) not confident or 1) confident. In addition, the presence of papillary muscle enhancement, RV scar, and LV thrombus were each assessed separately on a binary scale: 0) not present or 1) present. In a subgroup of 20 subjects, intra- and inter-observer agreement was assessed for both the presence of scar as well as total scar burden.

### Statistical analysis

All statistical analyses were performed using SPSS Statistics 23 (Statistical Package for the Social Sciences (SSPS), International Business Machines, Inc., Armonk, New York, USA). Results are expressed as mean ± standard deviation or as percentage unless otherwise specified. Differences in the detection of myocardial scar between conventional bright-blood LGE and dark-blood LGE were evaluated using McNemar tests. Differences in total scar burden as assessed by both methods were evaluated using either the paired-sample *t*-test (normally distributed data) or the non-parametric Wilcoxon signed-rank test (non-normally distributed data). Normality of data was confirmed using the Shapiro-Wilk test. Differences in image quality and observer confidence between both methods were evaluated using a Wilcoxon signed-rank test and the McNemar test, respectively. Intra- and inter-observer variability in the presence of scar was assessed by calculating Cohen’s kappa and Fleiss’ kappa coefficients, respectively. Intra- and inter-observer variability in the total scar burden was assessed by calculating the intraclass correlation coefficient. Coefficients were considered: ‘poor’ < 0.40, ‘fair’ between 0.40 and 0.59, ‘good’ between 0.60 an 0.74, and ‘excellent’ > 0.75. All statistical tests were two-tailed and *p*-values < 0.05 were considered significant.

## Results

### Study population

Baseline characteristics of the study population are summarized in Table 2. Complete conventional bright-blood and dark-blood LGE data sets were acquired in all subjects (*n* = 300). Eight subjects (2.7%) had non-diagnostic bright-blood and/or dark-blood LGE image quality due to arrhythmia (*n* = 1), image artefacts (including respiratory motion, wrap-around, and shading) (*n* = 6), or a combination of both (n = 1), and were excluded from further analysis. Exclusion of these eight patients was caused by non-diagnostic image quality in the bright-blood LGE images only (*n* = 4), dark-blood LGE images only (*n* = 1), or both sets of images (*n* = 3). Statistical analysis was therefore performed on a population of 292 subjects. In total 4964 segments were analysed for LGE.

**Table 2:** Baseline characteristics of study population (*n* = 300)

*Age (years)*	55 ± 16
*Gender (male, %)*	179 (59.7%)
*Weight (kg)*	83 ± 20
*Length (cm)*	171 ± 10
*BMI (kg / m* ^*2*^ *)*	28.2 ± 5.9
*BSA (m* ^*2*^ *)*	2.0 ± 0.3
*LVEDVi (mL/m* ^*2*^ *)*	92 ± 30
*LVESVi (mL/m* ^*2*^ *)*	48 ± 30
*LVSVi (mL/m* ^*2*^ *)*	44 ± 10
*LVEF (%)*	51 ± 14
*RVEF (%)*	55 ± 10
*Known significant CAD (n, %)*	68 (22.7%)
*Previous myocardial infarction (n, %)*	48 (16.0%)
*Previous PCI (n, %)*	32 (10.7%)
*Previous CABG (n, %)*	10 (3.3%)
*Indication for CMR (n, %)*	
*Cardiomyopathy*	172 (57.3%)
*Myocardial infarction / viability*	52 (17.3%)
*Myocarditis*	33 (11.0%)
*Aortic pathology*	3 (1.0%)
*Valvular disease*	2 (0.7%)
*Pericardial disease*	3 (1.0%)
*Cardiac mass*	1 (0.3%)
*Stress perfusion*	102 (34.0%)
*Final diagnosis (n, %)*	
*Normal*	93 (31.0%)
*Non-ischemic cardiomyopathy*	80 (26.7%)
*Myocardial infarction*	87 (29.0%)
*Ischemia*	34 (11.3%)
*Myocarditis*	17 (5.7%)
*Aortic pathology*	2 (0.7%)
*Valvular disease*	4 (1.3%)
*Pericardial disease*	4 (1.3%)
*Cardiac mass*	0 (0%)
*Other*	1 (0.3%)

Values are n, mean ± standard deviation, or frequency (%). Subjects may be known with multiple indications for CMR and/or multiple final diagnoses

*BMI* body mass index, *BSA* body surface area, *CABG* coronary artery bypass graft, *CAD* coronary artery disease, *CMR* cardiovascular magnetic resonance, *LVEDVi* indexed left-ventricular end-diastolic volume, *LVEF* left-ventricular ejection fraction, *LVESVi* indexed left-ventricular end-systolic volume, *LVSVi* indexed left-ventricular stroke volume, *PCI* percutaneous coronary intervention, *RVEF* right-ventricular ejection fraction

### LV scar detection

**Fig. 2:**
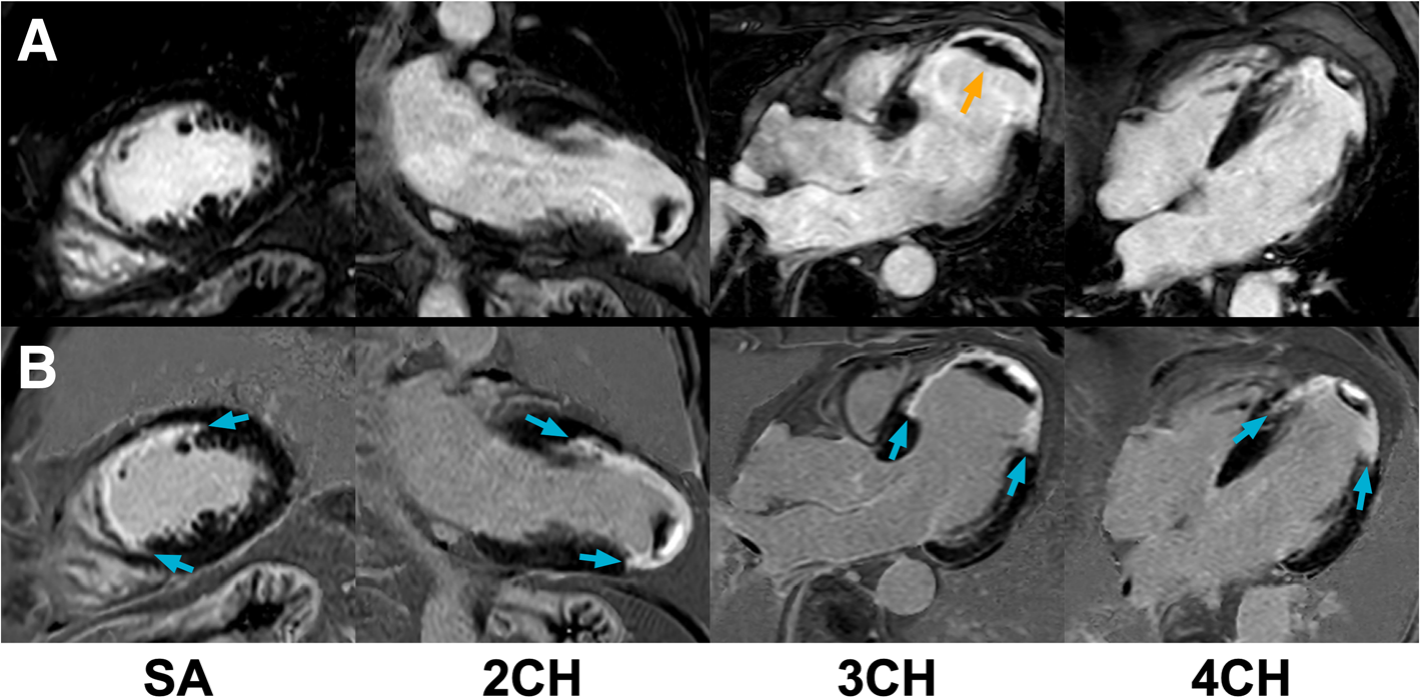
Bright-blood and dark-blood LGE images of a subject with myocardial infarction and a large apical thrombus. *Panel* ***a****:* Conventional bright-blood phase sensitive inversion recovery (PSIR) LGE images of the short-axis at mid-ventricular level (SA), two-chamber view (2CH), three-chamber view (3CH), and four-chamber view (4CH), which show a myocardial infarction in the left anterior descending (LAD)-territory with transmurality ranging from 75 to 100% and a large area of left ventricular (LV) thrombus in the apex (orange arrow). *Panel* ***b****:* Dark-blood PSIR LGE images of the same views. Although the myocardial infarction was clearly seen by both LGE methods, the transmural extent was challenging to assess on the bright-blood LGE images due to poor definition of the border between scar and LV blood. In contrast, both the short-axis view and long-axis views obtained by dark-blood LGE allowed clear delineation of the (transmural extent of the) infarcted area (blue arrows). Additionally, the area of LV thrombus is still clearly visible due to the dark-blood effect (blood appears dark gray) rather than a black-blood effect. In this case, conventional LGE and dark-blood LGE were performed at 10 min and 20 min post-injection, respectively. For specific scan details, see ‘Philips Ingenia’ at Table 1

**Fig. 3:**
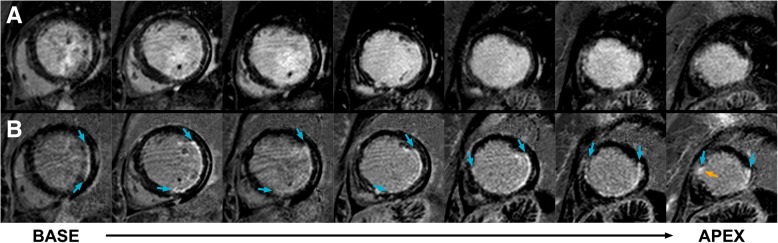
Short-axis bright-blood and dark-blood LGE images of a subject with myocardial infarction. *Panel* ***a****:* Conventional bright-blood phase sensitive inversion recovery (PSIR) LGE images of the short-axis from base to apex, which show a subendocardial infarction of the basal and mid inferolateral and lateral wall, and near-transmural infarction of the apical lateral wall. The short-axis views obtained by bright-blood LGE could not accurately visualize the transmural extent of the myocardial infarction *Panel* ***b****:* Dark-blood PSIR LGE images of the same views. In contrast to conventional bright-blood LGE (as shown in panel **a**), the short-axis views obtained by dark-blood LGE allowed clear delineation of the myocardial infarction (blue arrows). Furthermore, focal subendocardial LGE was detected in the apical septum (orange arrow), which was missed using bright-blood LGE only. Please note the presence of minor image artefacts caused by respirational motion, which are not inherently related to the proposed dark-blood LGE and are visible on both conventional as well as dark-blood LGE images. In this case, conventional LGE and dark-blood LGE were performed at 10 min and 20 min post-injection, respectively. For specific scan details, see ‘Philips Achieva TX’ at Table 1

**Fig. 4:**
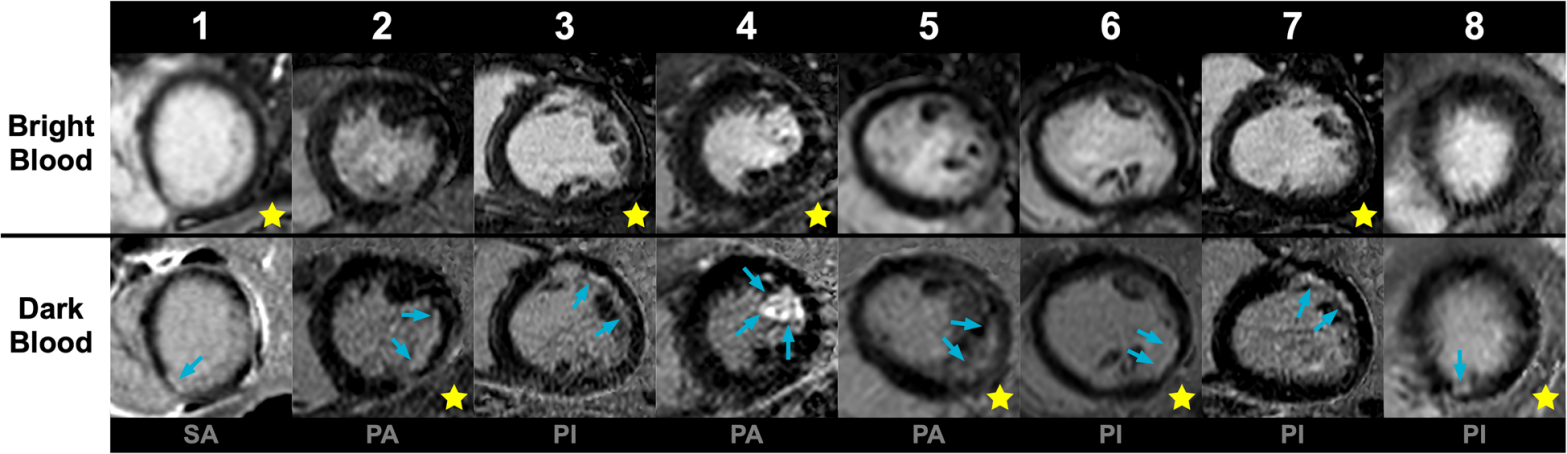
Short-axis bright-blood and dark-blood LGE image of all eight discordant cases. Conventional bright-blood (upper row) and dark-blood (lower row) phase sensitive inversion recovery (PSIR) LGE short-axis images of all eight discordant cases. Areas of infarction are indicated by blue arrows. Note that these areas of infarction could have been recognized using conventional bright-blood LGE when the dark-blood images would have been shown simultaneously. However, when only the conventional bright-blood images were shown, these areas were missed and the patients were declared free of any ischemic scar, while dark-blood LGE showed clear hyperenhancement. Patient 2, 3, 4, 5, 6, and 8 had known coronary artery disease. In each patient, the yellow star indicates which method was performed first (at 10 min), while the two letters indicate the CMR system used for each study (see Table 1 for specific scan details, PI = Philips 1.5T Ingenia, SA = Siemens 1.5T Aera, PA = Philips 3T Achieva TX)

**Fig. 5:**
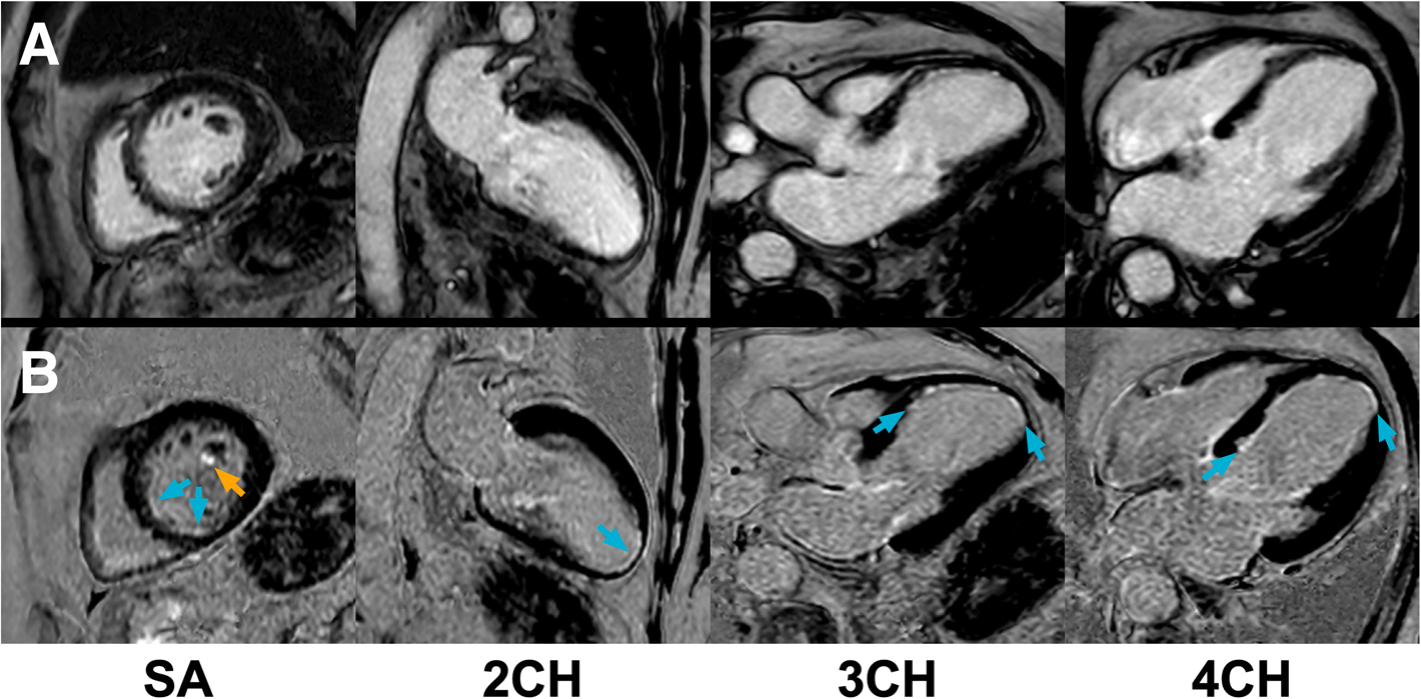
Bright-blood and dark-blood LGE images of a subject with thin subendocardial infarction. *Panel* ***a****:* Conventional bright-blood phase sensitive inversion recovery (PSIR) LGE images of the short-axis at mid-ventricular level (SA), two-chamber view (2CH), three-chamber view (3CH), and four-chamber view (4CH), which show a possible subendocardial infarction in the LAD territory. The transmurality of the scar is not well defined. *Panel* ***b****:* Dark-blood PSIR LGE images of the same views reveal a 25% subendocardial myocardial infarction (blue arrows) in the septal segments and 25–50% transmurality in the apical lateral wall. Furthermore, the short-axis dark-blood LGE image shows enhancement of the anterolateral papillary muscle (orange arrow), which was not observed using conventional bright-blood LGE. In this case, conventional LGE and dark-blood LGE were performed at 20 min and 10 min post-injection, respectively. For specific scan details, see ‘Philips Ingenia’ at Table 1

**Fig. 6:**
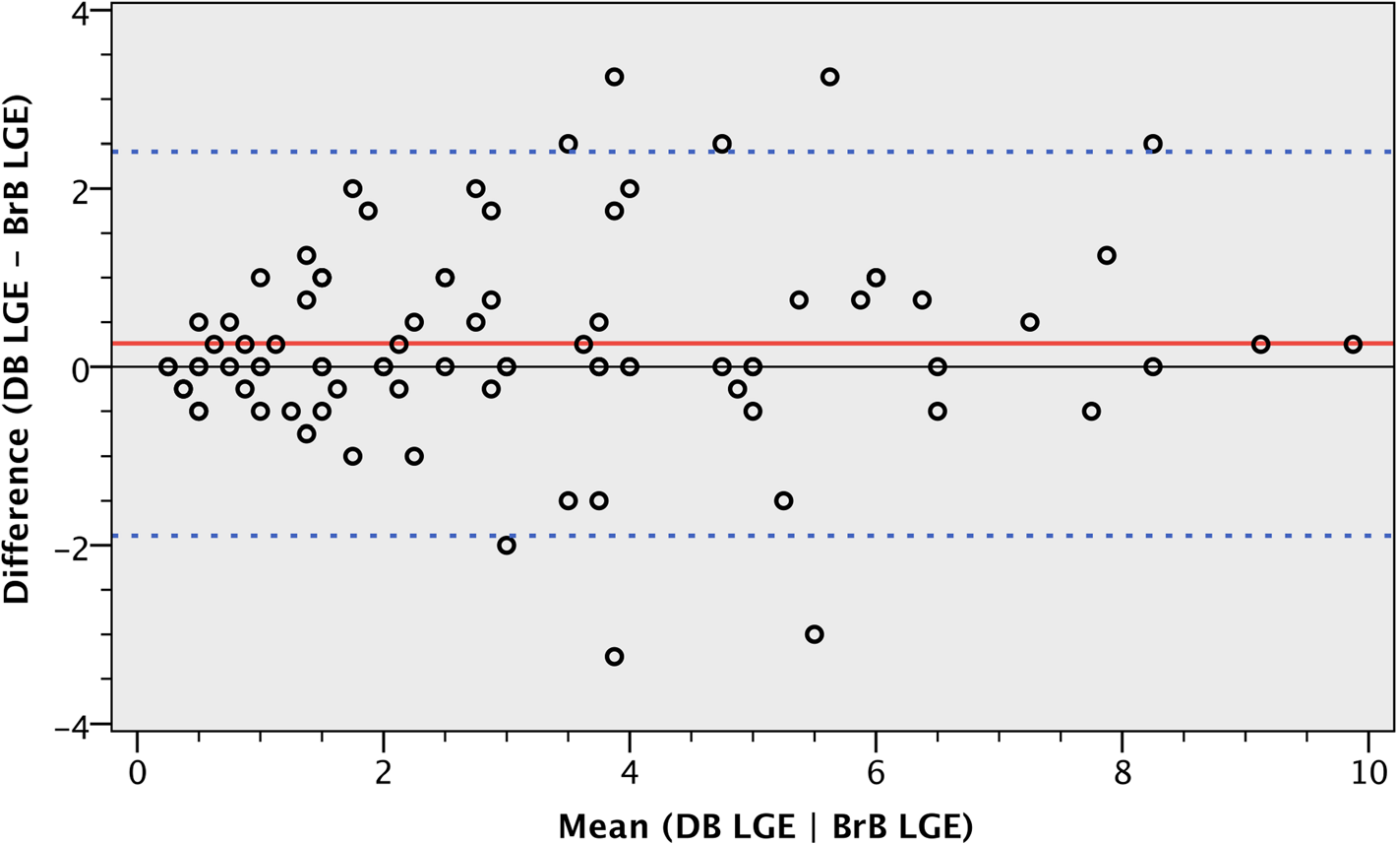
Bland-Altman plot of total scar burden as assessed by both conventional and dark-blood LGE. Mean total scar burden and difference in total scar burden are illustrated for all 89 cases that showed ischaemic scar on both conventional bright-blood LGE and dark-blood LGE. A significant bias (red solid line) of + 0.3 for dark-blood LGE was observed, with limits of agreement (blue dashed lines) at − 1.9 and + 2.4

**Fig. 7:**
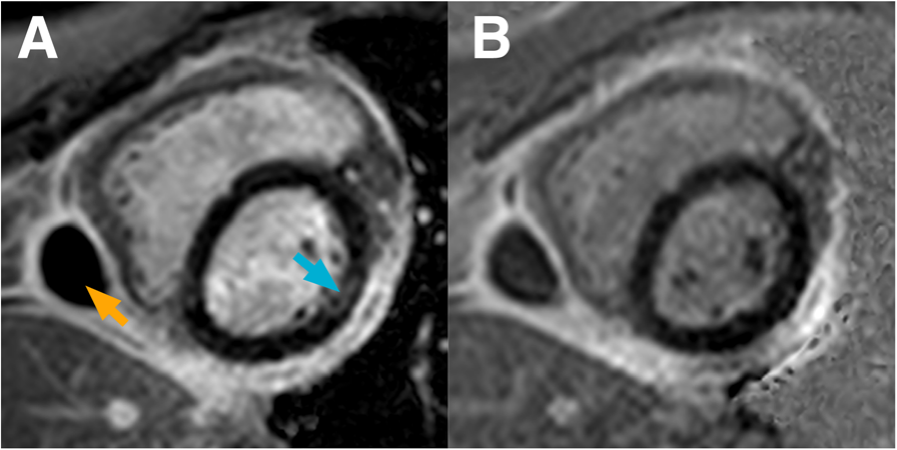
Short-axis bright-blood and dark-blood phase sensitive inversion recovery (PSIR) LGE image of a subject with non-ischaemic fibrosis. *Image* ***a****:* Conventional bright-blood PSIR LGE short-axis image demonstrating the presence of mid-myocardial enhancement in the basal to mid antero-lateral segment (blue arrow). The orange arrow indicates a pericardial cyst/loculated pericardial effusion in the context of thickened pericardium. In this case, a diagnosis of previous myo-pericarditis was made on the basis of CMR findings and clinical history. *Image* ***b****:* No evidence of mid-myocardial enhancement was found on the dark-blood PSIR LGE image. A non-perfect correspondence of the slice positioning, most likely due to patient motion between acquisitions, could have contributed to this discrepancy. In this case, conventional LGE and dark-blood LGE were performed at 10 min and 20 min post-injection, respectively. For specific scan details, see ‘Philips Achieva TX’ at Table 1

An ischemic/subendocardial LV scar pattern was detected in 89 subjects by bright-blood LGE and in 97 subjects by dark-blood LGE (30.5% vs 33.2%, *p* = 0.008). All subjects with a definite diagnosis of ischemic scar on bright-blood LGE were also correctly identified by dark-blood LGE imaging (Figs. 2 and 3). However, eight subjects (8.3%) were declared free of any ischemic scar using conventional bright-blood LGE, but showed subendocardial enhancement using dark-blood LGE (Fig. 4). This effect was observed regardless of which sequence was acquired first (4 cases for each order of acquisition), and of the type of scanner used (Siemens 1.5T Aera: *n* = 1; Philips 1.5T Ingenia  T: *n* = 4; Philips 3T Achieva: *n* = 3). Six out of these eight subjects were already known with ischemic heart disease (myocardial infarction [*n* = 2], previous percutaneous coronary intervention [n = 2], previous coronary artery bypass graft [n = 1], or a combination of myocardial infarction and revascularization [n = 1]). Total scar burden in subjects with ischemic scar was significantly higher on dark-blood LGE images compared to bright-blood LGE images (3.3 ± 2.4 vs 3.0 ± 2.3 standard AHA segments, *p* = 0.015; Figs. 5 and 6). Non-ischemic scar patterns were observed in 46 and 44 subjects with bright-blood LGE and dark-blood LGE, respectively (15.8% vs 15.1%). Although areas of non-ischemic scar were missed in two cases using dark-blood LGE in the present study (Fig. 7), interestingly, no cases of non-ischemic scar were missed when dark-blood LGE was performed at 10 min post-injection.

### Detection of papillary muscle enhancement, RV scar, and areas of thrombus

Papillary muscle enhancement was detected in 17 (19.1%) subjects with ischaemic scar using conventional bright-blood LGE and in 34 (35.1%) subjects with ischaemic scar using dark-blood LGE. RV infarction/fibrosis was detected in 6 and 7 cases using bright-blood LGE and dark-blood LGE, respectively. The single case that was detected by dark-blood LGE only, was later diagnosed with arrhythmogenic RV cardiomyopathy (ARVC). No difference in the detection of LV thrombus was observed between both LGE methods (10 cases in total, Fig. 2).

### Overall image quality

**Fig. 8:**
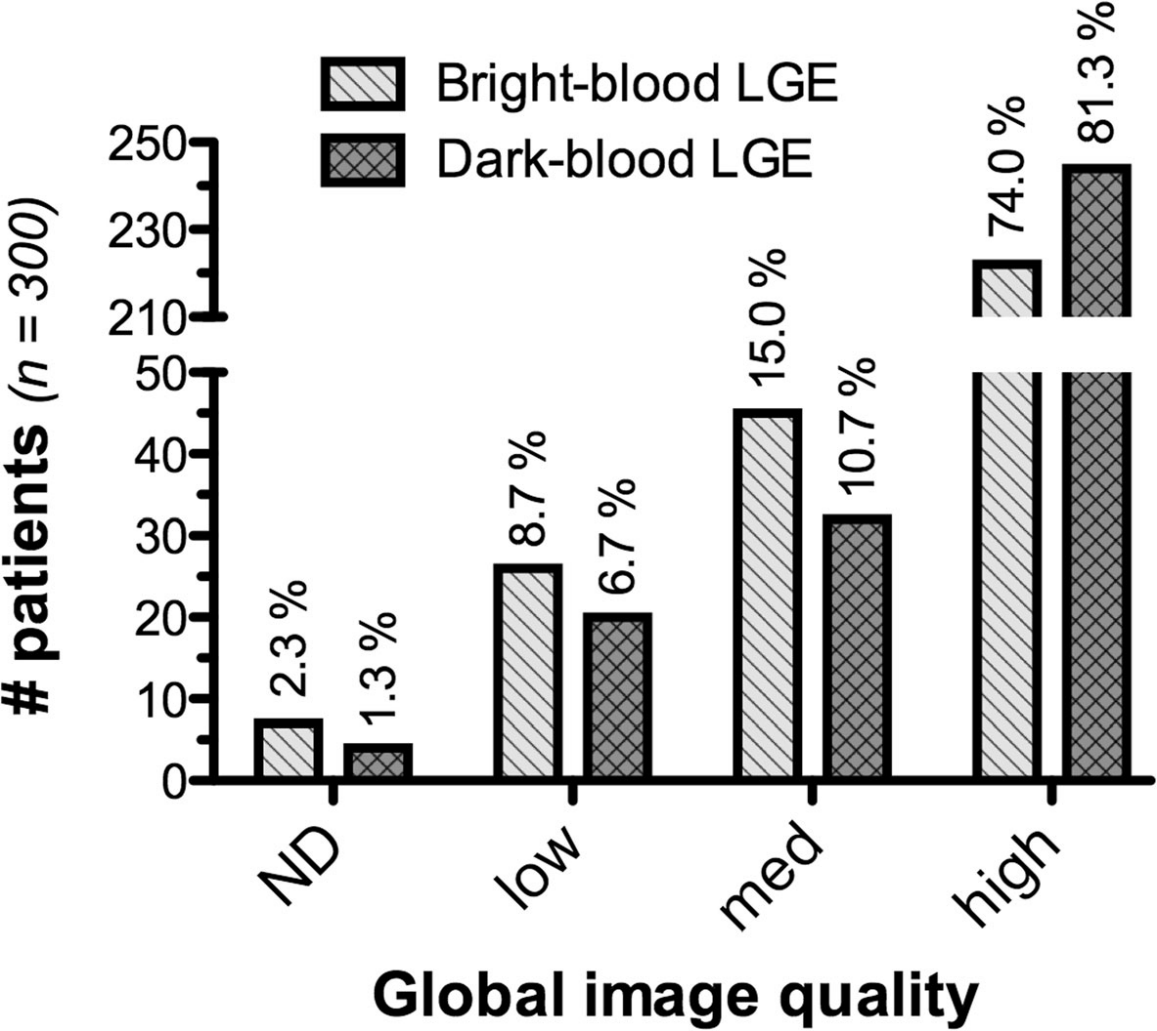
Overall image quality of bright-blood and dark-blood LGE images. Overall image quality was scored as: 0) non-diagnostic, 1) low [≥ two segments non-diagnostic], 2) medium [one segment non-diagnostic], or 3) high [all segments correctly identified]. The exact number of subjects is given using the vertical axis, while percentages of the study population (*n* = 300) are indicated above each bar. Note that eight subjects had non-diagnostic image quality and were excluded for further analysis

Overall image quality for the entire study population (*n* = 300) is illustrated in Fig. 8. Image quality was significantly better using dark-blood LGE than conventional bright-blood LGE (*p* = 0.006). All LV segments were of the highest diagnostic quality in 74.0% vs 81.3% of the subjects using conventional bright-blood and dark-blood LGE, respectively. Non-diagnostic image quality was observed in 7 (2.3%) and 4 (1.3%) cases using conventional bright-blood LGE and dark-blood LGE, respectively, which led to the exclusion of 8 cases in total. In the subgroup where dark-blood LGE was performed first at 10 min post-injection, no significant difference in overall image quality was found between the two methods (*p* = 0.563). However, when conventional bright-blood was performed first, dark-blood LGE showed significantly higher overall image quality (*p* = 0.002).

### Observer confidence

Significantly higher observer confidence was found using dark-blood LGE compared to conventional bright-blood LGE (*p* = 0.033). Observers were confident in in 78.4% vs 84.2% of the subjects (*n* = 292) using conventional bright-blood and dark-blood LGE, respectively. In the subgroup where dark-blood LGE was performed first at 10 min post-injection, no significant difference in observer confidence was found between the two methods (*p* > 0.999). However, when conventional bright-blood was performed first, dark-blood LGE showed significantly higher observer confidence (*p* = 0.005).

### Intra- and inter-observer agreement

Complete intra-observer agreement in the presence of scar was observed for both conventional bright-blood LGE and dark-blood LGE (κ = 1.00). For the inter-observer variability in the presence of scar, excellent agreement was found for both conventional bright-blood LGE (κ = 0.90) and dark-blood LGE (κ = 0.85). For the assessed total scar burden, excellent intra-observer agreement was found for both conventional bright-blood LGE (ICC = 0.95) and dark-blood LGE (ICC = 0.99). For the inter-observer variability in total scar burden, excellent agreement was found for both conventional bright-blood LGE (ICC = 0.77) and dark-blood LGE (ICC = 0.88).

## Discussion

The aim of this study was to assess the clinical value of a novel dark-blood LGE approach, which does not require any additional magnetization preparation, in a large unselected cohort of 300 consecutive patients on multiple scanners with varying field strengths and from different vendors. This study shows that dark-blood LGE is more sensitive than conventional bright-blood LGE in the detection of ischemic scar, regardless of the field strength or scanner vendor, with higher average scar burden, increased overall image quality, and improved observer confidence. Dark-blood LGE led to the detection of ischemic scar patterns in subjects that were deemed free of scar by conventional bright-blood LGE, which has implications for patient management through the appropriate administration of secondary preventive therapies. Furthermore, no cases of LV thrombus were missed by dark-blood LGE in comparison to conventional bright-blood LGE.

Accurate subendocardial scar detection and delineation is crucial in patients with previous MI, as current treatment strategies are based on this assessment and the presence of even small areas of myocardial scar are prognostically significant [3, 4]. Conventional bright-blood LGE using myocardium nulling is not always able to detect these areas immediately at 10 min after injection. As the contrast between subendocardial scar and the blood pool depends on a number of variables including gadolinium clearance, performing conventional bright-blood LGE later after contrast injection will readily increase subendocardial conspicuity [30]. However, even in the subgroup where bright-blood LGE was performed later and was expected to have superior contrast, dark-blood LGE (already performed at 10 min post-injection) showed increased subendocardial scar detection, potentially increasing the efficiency of clinical protocols.

Other solutions to improve scar-to-blood contrast for better subendocardial scar conspicuity include numerous recently proposed methods that use additional magnetization preparation mechanisms to suppress LV blood pool signal [18–26]. However, a potential disadvantage of these dark- and black-blood methods is the decrease in scar-to-myocardium contrast, potentially compromising the detection of non-ischemic scar compared to bright-blood LGE [31]. In contrast, dark-blood LGE without magnetization preparation is able to maintain, or even exceed, the scar-to-myocardium contrast of conventional LGE, in addition to its superior scar-to-blood contrast [32].

Another important feature to consider when assessing the diagnostic capabilities of the proposed dark-blood LGE method is the accuracy of LV thrombus detection. Areas of LV thrombus can frequently be found in dysfunctional ventricles, most likely in correspondence with myocardial scar and wall motion abnormalities. When not identified and treated correctly, they can potentially result in life-threatening complications such as embolic myocardial infarction or cerebrovascular accident. As thrombus is characterized by its black appearance, conventional bright-blood LGE (or preferably early gadolinium enhancement) provides excellent contrast between the bright-blood and the black thrombus. Recently, various black-blood LGE approaches have been proposed that completely null the blood signal to improve subendocardial scar detection. As blood is rendered completely black using these sequences, thrombus may then seem to be part of the blood pool. However, by causing a dark-blood effect rather than a complete black-blood effect, blood will appear dark-gray and still allows the visualization of areas of thrombus with similar accuracy as conventional bright-blood LGE (Fig. 2) [27].

This study illustrated the clinical performance of a novel dark-blood LGE approach against conventional bright-blood LGE in a real-world situation. A large unselected cohort of 300 consecutive patients referred for clinical CMR including LGE was used, representing a typical everyday non-congenital case mix of ischemic and non-ischemic cardiomyopathies. The improved detection of ischemic scar, as well as the reliable detection of non-ischemic scar, RV scar, and LV thrombus, all achievable at 10 minutes post-injection, make the proposed dark-blood LGE method a viable and time-efficient alternative to conventional bright-blood LGE. These findings are of high applicability in most routine clinical settings, as the proposed dark-blood LGE approach, in contrast to other recently proposed dark- and black-blood techniques, is readily available and does not require any scanner adjustments and/or extensive optimizations.

### Limitations

Although the proposed dark-blood LGE method shows improved ischemic scar detection compared to conventional bright-blood LGE in a large cohort of patients, there was no histological confirmation. Importantly however, 75% of the subjects who showed an ischemic scar pattern on dark-blood LGE but not on bright-blood LGE, had known coronary artery disease that was related to the infarct territory. Whereas the authors feel the outcomes to be related to the proposed dark-blood LGE mechanism without using additional magnetization preparation, future studies that use histological validation in preclinical models would be of great value.

As the appearance of the conventional bright-blood and dark-blood LGE images are intrinsically different, full blinding for image type was impossible for this study. However, all LGE images were separated, anonymized, and analysed in random order at different timepoints. Thereby, conventional bright-blood and dark-blood LGE images were never presented in sequence to the reader. Moreover, the readers were not informed of the aim of the study or of the technical details of the sequences acquired. Therefore, all readers were, as far as possible, blinded for image type in this study.

Although we also report on the detection of non-ischaemic fibrosis, papillary muscle enhancement, RV fibrosis, and areas of LV thrombus, it should be stressed that the present study was not designed to draw any conclusions on these findings and therefore no *p*-values were provided. Moreover, as many discrete patterns of non-ischemic fibrosis are described in this general referral cohort, the present study would be underpowered to do so. As the quality of novel dark- and black-blood techniques is much debated for the detection of non-ischemic scar, future studies should focus on a comparison with conventional LGE regarding this type of scarring. Even though no conclusions are drawn on papillary muscle enhancement, the prevalence of papillary muscle enhancement in patients with MI in our study population is in agreement with values from previous literature [33].

Finally, signal threshold-based analyses have been used for LGE to delineate regions of scar. Exploring the impact of the dark-blood LGE approach used in the present study on signal threshold-based analyses was considered out-of-scope, but may be of interest for future studies.

## Conclusions

Dark-blood LGE is more sensitive for the detection of ischemic scar than conventional bright-blood LGE, on both 1.5 T and 3 T MR scanners of different vendors, without compromising the detection of non-ischemic scar and areas of thrombus. Furthermore, dark-blood LGE showed higher average scar burden, increased overall image quality, and improved observer confidence. As all these features are already achievable at 10 minutes after contrast administration, this novel dark-blood LGE approach emerges as a valuable and time-efficient diagnostic tool in the non-invasive assessment of myocardial scar. The applicability in routine clinical practice is further strengthened, as this approach is readily available without the need for scanner adjustments and/or extensive optimizations.

## Data Availability

The acquired datasets used in this study are available from the corresponding author on reasonable request.

## Abbreviations

2CH: Two chamber
3CH: Three chamber
4CH: Four chamber
AHA: American Heart Association
ARVC: Arrhythmogenic right ventricular cardiomyopathy
CMR: Cardiovascular magnetic resonance
LAD: Left anterior descending
LGE: Late gadolinium enhancement
LV: Left ventricle/left ventricular
MI: Myocardial infarction
PSIR: Phase sensitive inversion recovery
RV: Right ventricle/right ventricular
SA: Short axis
SCMR: Society for Cardiovascular Magnetic Resonance
TI: Inversion time
